# Sensory Outcomes and Neurotization Techniques Following Mastectomies: A Comprehensive Systematic Review

**DOI:** 10.3390/cancers18071052

**Published:** 2026-03-24

**Authors:** Beryl Zhou, Denis Cipurko, Rebeka Dejenie, Maeson Zietowski, Daniel Wong, Summer E. Hanson

**Affiliations:** 1 Pritzker School of Medicine, University of Chicago, Chicago, IL 60637, USA; 2 Section of Plastic and Reconstructive Surgery, University of Chicago Medicine and Biological Science Division, Chicago, IL 60637, USA

**Keywords:** breast reconstruction, neurotization, nerve coaptation, autologous, implant-based reconstruction, sensory recovery

## Abstract

Background/Objectives: With increasing survival rates following oncologic mastectomies, loss of breast sensation can negatively impact a patient’s quality of life. Methods: PubMed, Embase, and Web of Science were searched in April 2025 for studies reporting sensory outcomes after neurotized breast reconstruction. Eligible studies included patients undergoing autologous or implant-based reconstruction with any neurotization technique. Forty studies were included, and outcomes involved objective sensory testing (e.g., Semmes-Weinstein monofilaments, pressure-specified sensory devices, and thermal thresholds) and patient-reported quality of life (e.g., BREAST-Q). Results: Neurotization consistently accelerated and improved recovery of tactile, thermal, and protective sensation compared with non-neurotized controls, particularly in DIEP and TRAM flaps. Direct coaptation was most frequently employed, while nerve allografts, conduits, and autologous grafts offered effective alternatives when direct repair was not feasible. Implant-based reconstructions using allografts also demonstrated significant improvements in the nipple–areola complex and breast skin sensation. Across studies, earlier and more uniform sensory return was reported, with improved sensation often associated with high patient satisfaction and quality of life. Conclusions: The preponderance of observational evidence suggests that nerve coaptation, whether by direct suture, conduit, allograft, or autograft, represents a promising adjunct to breast reconstruction in both autologous and implant-based reconstruction. However, many studies were retrospective in design, had small sample sizes, and lacked randomization.

## Simple Summary

Breast sensation is often lost at the time of mastectomy and has a significant impact on a patient’s quality of life and survivorship. New approaches seek to restore sensation in the setting of breast reconstruction. This review summarizes the current body of evidence for implant-based reconstruction as well as tissue-based reconstruction, and illustrates the need for longer follow-up and standardized outcome measures. Still, the opportunity to improve post-mastectomy numbness or pain should continue to be explored.

## 1. Introduction

Breast cancer is the most common cancer type among women, with a lifetime risk of 12.9% for women born in the United States [1]. Disease prognosis has significantly improved over time, with 5-year survival rates reaching ranges of 90% and 10-year survival rates at about 80% [2]. An increasing number of cured patients also represent a higher demand for breast-conserving therapies alongside mastectomy with breast reconstruction [3,4,5].

During the mastectomy procedure the lateral and anterior intercostal nerves are typically transected, resulting in a loss of sensation after mastectomy. In some instances, spontaneous reinnervation will occur as a result of peripheral migration [6]. Because loss of sensation can negatively affect patient satisfaction, neurotization techniques in breast reconstruction can offer substantial improvements in a patient’s quality of life and well-being [7]. Slezak et al. first demonstrated sensory restoration in autologous tissue transfers by coapting intercostal nerves during a TRAM reconstruction in 1992, which challenged the focus on aesthetic outcomes alone [8]. Over the following decades, techniques have evolved to improve sensation and bridging nerve gaps in implant-based reconstruction.

Various techniques for breast reconstruction can be categorized based on whether the reconstructed breast mound is implant-based (using saline or silicone devices) or autologous (using one’s own tissue), such as those from the lower abdomen in the deep inferior epigastric perforator (DIEP) flap or the transverse rectus abdominis myocutaneous (TRAM) flap. Other less common sources include the lateral thigh perforator flap (LTP), the latissimus dorsi (LD) flap, the profunda artery perforator (PAP) flap from the upper thigh, and the superior/inferior gluteal artery perforator (SGAP/IGAP) flap from the buttocks [9]. For neurotization, a recipient third or fourth intercostal nerve (ICN) on the chest wall is typically identified and preserved, along with a donor sensory nerve within the flap (most commonly T10–T12 thoracoabdominal branches of a DIEP or TRAM) [10]. The recipient and donor nerves can be connected via direct coaptation, or aided by a conduit, allograft, or autologous nerve graft.

Implant-based reconstruction is more commonly performed worldwide and may be staged using a tissue expander or a single direct-to-implant technique [11]. Sensory reinnervation with this technique typically involves preserving one or more intercostal nerves (T3–T5) during nipple-sparing mastectomy (NSM) and bridging them to recipient targets with decellularized cadaveric nerve allografts, autologous grafts (adjacent intercostal or sural nerve), or synthetic/biocompatible nerve conduits [9,12].

Recovery following a nerve injury is affected by several factors, including the interval between the injury and treatment, the patient’s age, the presence of associated soft tissue or vascular damage, and the proximity of the lesion site to the structures it supplies [13]. A tension-free coaptation with epineural sutures to reduce adhesions and scar formation is the standard practice, but it is not always possible when there are significant nerve gaps [14]. For direct end-to-end repair, sutures are placed into the epineurium with 9-0 or 10-0 nylon that are used to avoid malrotation of the nerve ends [15]. Primary or immediate repair usually takes place at the time of mastectomy, whereas delayed repair takes place after the initial mastectomy.

The gold standard technique for coaptation is typically end-to-end neurorrhaphy between the proximal and distal nerve ends when no gap exists [16]. End-to-side is an alternative method in cases where the proximal nerve stump is inaccessible, and therefore the injured distal stump is coapted to the side of an uninjured donor nerve [14]. Sensory sprouting recovery is generally easier than motor sprouting for this technique, and can be used in noncritical sensory deficits [14,17,18].

Sensory outcomes can be measured objectively through pressure, pain, and temperature thresholds as well as subjectively from patient questionnaires. Quantitative sensory tests (QSTs) comprise static tests that assess response to a fixed stimulus (thermal pain thresholds, 2-point discrimination, pressure pain thresholds), or dynamic tests that assess the sensory system’s changes in response to stimuli over time (conditioned pain modulation, temporal summation, etc.) [19]. While sensation testing methods have been widely variable, the most used metrics include the pressure specified sensory device (PSSD; AxoGen, Alachua, FL, USA), Semmes-Weinstein monofilaments (SWM), two-point discrimination (Disk-Criminator, US Neurologicals, Poulsbo, WA, USA), and temperature thresholds. For patient experience, BREAST-Q is commonly used to evaluate the impact of surgery on a patient’s health-related quality of life and overall well-being.

Previous systematic reviews have determined that earlier, more uniform, and better overall sensory recovery occurs in innervated breasts compared to non-innervated breasts [20]. Shiah et al. found that pooled neurotization success rates were up to 90.6% (95% CI: 83.6–96.0%) in the nine studies that reported their attempts. Since its publication in 2022, many more studies have been performed to evaluate emerging strategies involving nerve allografts, autologous grafts, and hybrid approaches for implant-based methods. With breast cancer survivorship increasing, patient priorities have extended to include sensory rehabilitation as a factor linked to quality of life. By synthesizing the current evidence, this review aims to evaluate emerging neurotization techniques and understand factors influencing sensory recovery.

## 2. Materials and Methods

A comprehensive literature search was conducted on 9 April 2025 to identify all studies reporting outcomes of neurotized breast reconstruction adhering to the Preferred Reporting Items for Systematic Review and Meta-Analysis (PRISMA) statement guidelines and has not been registered [21]. The PubMed (National Library of Medicine, Bethesda, MD, USA), Embase (Elsevier, Amsterdam, Netherlands), and Web of Science (Clarivate Analytics, Philadelphia, PA, USA) databases were searched using key terms involving neurotization, sensation, and breast reconstruction (Supplemental Materials: Data research, Tables S1 and S2).

The primary objective of this systematic review was to evaluate whether neurotization in breast reconstruction improves objective sensory recovery compared to non-neurotized reconstruction following oncologic or risk-reducing mastectomy. Primary endpoints were objective measures of sensory recovery, including pressure thresholds measured by SWM, static and dynamic thresholds measured by PSSD, two-point discrimination thresholds, and thermal or pain threshold testing. Secondary objectives included comparing outcomes across different neurotization techniques (direct coaptation, nerve allograft, autograft, etc.), comparing sensory outcomes between autologous and implant-based reconstruction, and evaluating patient-reported quality-of-life outcomes associated with sensory recovery. These endpoints included patient-reported outcomes (assessed using the BREAST-Q instrument), domain-specific quality-of-life measures (psychosocial, sexual, and physical well-being), operative time associated with neurotization, and postoperative complications.

**Figure 1:**
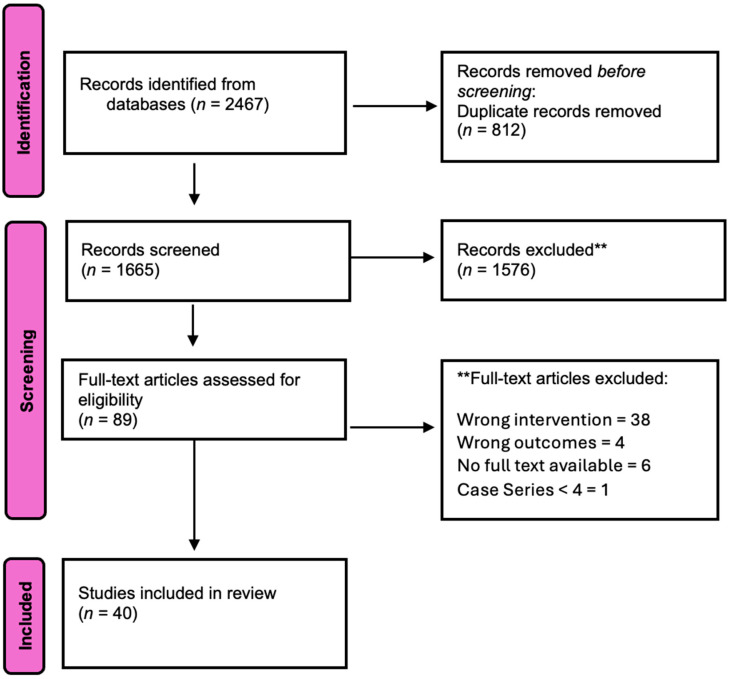
PRISMA flow diagram. This includes the search and screening results of the systematic literature review.

Studies were included if they met the following criteria: reported sensory outcomes following neurotized breast reconstruction, included patients undergoing autologous or implant-based reconstruction after oncologic or prophylactic mastectomy, evaluated any neurotization technique, reported objective sensory testing and/or patient-reported outcomes, included >4 patients, and were published in English with full-text availability. Studies were excluded if they involved cosmetic breast surgery or gender-affirming procedures, did not report measurable sensory outcomes, lacked sufficient methodological data, or were review articles, editorials, case reports, or case series with fewer than four patients. Additionally, we explored potential modifying factors that influenced sensory recovery, such as radiation therapy, reconstruction timing, and donor nerve selection. A total of 2467 articles from PubMed, Embase, and Web of Science were identified, 812 were removed as duplicates. Two reviewers (BZ and DC) independently screened titles and abstracts, followed by full-text screening by two other independent reviewers (MZ and RD). Discrepancies at any stage were resolved through discussion or consultation with a third reviewer. Ultimately, 40 studies were included in the final analysis. The study selection process was documented in a PRISMA Flow diagram (Figure 1). Data were then extracted by two independent reviewers, including: study characteristics (author, year, design, country), participant details (number of patients, mean age, mean BMI), reconstruction details and timing, neurotization technique, outcomes, and overall findings. Risk-of-bias assessments were done by two independent reviewers (BZ and SEH) according to the Newcastle–Ottawa Scale (NOS) for non-randomized studies and the Cochrane Collaboration’s tool for randomized trials (Supplemental Table S1) [22,23]. Most non-randomized studies scored 5–6 out of 9, reflecting adequate cohort selection and generally reliable outcome assessments, but limited control for confounding. The most common sources of bias were non-randomized treatment allocation, small sample sizes, variable follow-up, and incomplete adjustment for baseline differences. Among the randomized studies, risk of bias was lower. Meta-analysis/quantitative pooling was not done due to substantial clinical and methodological heterogeneity in outcome reporting, including variation in testing modalities, reporting scales, anatomical testing zones, and follow-up durations. Future studies should adopt standardized reporting frameworks to facilitate quantitative synthesis.

## 3. Results

Forty articles were included in the final analysis that investigated breast sensation following neurotization in patients who underwent oncologic mastectomy. Of the 40 articles reviewed, 35 investigated neurotization in autologous breast reconstruction, and 8 included neurotization in implant-based reconstruction. In autologous reconstruction, direct coaptation alone was the most common technique reported in 20 studies (57.1%), followed by direct coaptation with allografts in 8 (22.9%) and with a conduit in three (8.6%). Additionally, one study (2.9%) combined direct coaptation with both allograft and autograft, another (2.9%) with allograft and conduit, and one (2.9%) compared direct coaptation alone versus with allograft. Two studies (5.7%) evaluated direct coaptation with autograft alongside implant-based neurotization with autograft. These studies are summarized in Table 1. For implant-based reconstruction, allografts were used in four studies (50.0%), autografts in two (25.0%), both in comparison with autologous techniques, and direct coaptation was evaluated in one (12.5%). These studies are summarized in Table 2.

### 3.1. Autologous Reconstruction: Direct Coaptation Only

Of the 40 studies included, 20 articles incorporating 774 patients evaluated sensory recovery following neurotization with direct coaptation. Most studies compared direct coaptation in neurotized reconstructed breasts to non-neurotized reconstructed breasts as controls, with the exception of 2 studies [24,25,26]. In Isenberg et al.’s studies, direct coaptation in TRAM was found to have earlier and more substantial sensory return compared to both neurotized LD and non-neurotized TRAM reported in the literature [25,26]. Additionally, Magarakis et al. found that neurotized DIEP flaps trended towards better static and moving sensation than non-neurotized DIEP flaps in cases without radiation therapy, but this was reversed in the case of irradiated cases [27]. However, the sample size was small and neurotization could not be evaluated as an independent predictor of sensation.

Modern sensory testing commonly reported using SWM, PSSD, and two-point discrimination tests in 9 standardized breast zones. In prospective studies comparing neurotized and non-neurotized DIEP flaps, direct coaptation provided tactile (SWM) and protective sensation, particularly in central and medial breast zones [28,29]. Beugels et al. reported that both immediate and delayed innervated DIEP flaps demonstrated improved sensation across all regions, as shown through lower monofilament values compared to non-innervated controls [30]. This was further supported by Bubberman et al., who noted that heat pain was often imperceptible in 42.1% of non-innervated flaps compared to 10.3% of innervated flaps [29]. Retrospective cohort studies conducted by Bijkerk et al. and Cornelissen et al. confirmed that innervated DIEP had better pressure sensation than non-innervated flaps, with longer follow-up times associated with improved recovery in both native breast skin and abdominal flap skin [31,32]. Prior studies support similar findings, with neurotized DIEP flaps having the highest sensory recovery to cold, warm, vibratory, and erogenous stimuli across the entire flap surface compared to non-neurotized DIEP and TRAM flaps [33]. Across these studies, no major complications were reported related to direct coaptation in autologous reconstruction, other than post-radiation fibrosis or short lengths of the donor or recipient nerves [31].

**Table 1:** Summary of studies evaluating sensory restoration of breast reconstruction with various neurotization techniques in autologous-based reconstruction.

Author	Year	Journal	Design	Recon Type	Follow Up Mean ± SD [Range]	Method	Sensory Testing	Donor Nerve	Recipient Nerve	Key Results
Zhang [24]	2025	Annals of Plastic Surgery	Retro	DIEP (+coap) (*n* = 11)	94.42 [71.98, 112.56];	Direct coap used end-to-end epineural coaptation with 9-0 nylon; allograft group used 70 mm Avance nerve allograft (Axogen, Alachua, FL, USA)	AcroVal PSSD (AxoGen)	Sensory branches of T10–12	Anterior cutaneous branch of the 3rd ICN	No significant difference in mean breast sensitivity between direct (64.58 g/mm^2^) and allograft (78.28 g/mm^2^) groups (*p* = 0.680); no regional sensory differences reached significance; BREAST-Q outcomes (psychosocial, sexual, satisfaction, physical, and sensation domains) were similar between groups (all *p* > 0.05); direct group had significantly longer follow-up for BREAST-Q (94.4 vs. 60.5 months, *p* = 0.013); both groups outperformed normative scores in multiple domains.
				DIEP (+allo) (*n* = 19)	60.45 [59.82, 70.34]					
Isenberg [25]	2004	Ann Plast Surg	Retro	TRAM (+) (*n* = 11) LD (+) (*n* = 4)	(2–16 months)	interrupted sutures; 10-0 nylon; microscope	SWM; 2-PD (Disk-Criminator); Sharp/dull (35G needle, cotton swab); Standard temp. probe	TRAM: intercostal perforator LD: thoracodorsal	Lateral ramus of 4th ICN	TRAM (+) flaps had greater pressure recovery than LD (+); both showed earlier sensory return than reported non-innervated cases and plateaued at 8–9 months; No difference in operative times and wound healing complications
Isenberg [26]	2002	J Reconstr Microsurg	Pros	pTRAM (+) (*n* = 10)	(2–25 months)	End-to end coap; microneurorrhaphy	SWM; 2-PD (Disk-Criminator); Sharp/dull (35G needle, cotton swab); Standard temp. probe	11th ICN	Lateral-anterior branch of 4th ICN	TRAM (+) flaps demonstrated early, progressive, and superior recovery compared with reported TRAM (−) cases, though no erogenous sensation returned
Magarakis [27]	2013	Microsurgery	Retro	Implant (−) (*n* = 20)	Median: 26 months (18–49)	End-to end coap	PSSD	Branch of iliohypogastric or nerve co-located with primary vascular perforator	-	In non-irradiated cases, implants showed better sensation than DIEP flaps, whereas irradiated DIEP flaps outperformed irradiated implants; DIEP (+) trended toward better sensation than DIEP (+) without adjuvant radiation, though conclusions were limited by small sample size; no return of erotic sensation.
				DIEP (−) (*n* = 12)	Median: 33 months (19–53)					
				DIEP (+) (*n* = 5)	-					
Beugels [28]	2021	Plast Reconstr Surg	Pros	DIEP (+) (*n* = 67) DIEP (−) (*n* = 58)	6 weeks, 3 months, 6–9 months, 12–15 months	End-to-end; microsurgical epineural coaptation with 9-0 nylon sutures	SWM	Anterior cutaneous sensory branch of the 10th–12th ICN	Anterior cutaneous branch of the 2nd or 3rd ICN	Innervated flaps had significantly better sensation in native, flap, and total skin (*p* ≤ 0.010); effect was stronger in immediate reconstructions; flap sensation improved more postoperatively in innervated group (*p* = 0.015–0.017); older age, higher flap weight, and shorter follow-up were associated with poorer sensory outcomes.
Bubberman [29]	2024	Breast Edinb Scotl.	Double-blind RCT	DIEP (+) (*n* = 19) DIEP (−) (*n* = 22)	24 months (interim analysis)	End-to-end; 9-0 nylon sutures; performed after vascular anastomosis with fibrin sealant; microsurgery	SWM, PSSD, and thermal stimulator (PATHWAY, Medoc Ltd., Israel)	Sensory branch of flap	Anterior cutaneous branch of 2nd or 3rd ICN	At 24 months, flap skin monofilament threshold was significantly lower in innervated vs. non-innervated flaps (4.48 vs. 5.20, *p* = 0.003); protective sensation was preserved more often in innervated flaps; PSSD thresholds were significantly better in the flap center (1-PS: 47.8 vs. 71.2 g/mm^2^, *p* = 0.036; 1-PM: 16.2 vs. 53.0 g/mm^2^, *p* < 0.001); heat pain was imperceptible in 42.1% of non-innervated vs. 10.3% of innervated flaps (*p* = 0.004)
Beugels [30]	2019	Plast Reconstr Surg	Pros	DIEP (−) (*n* = 45)	Median: 17 months (IQR 12–24)	End-to-end; 9-0 nylon; 2 stitches	SWM	Sensory cutaneous branch of 10th–12th ICN running with perforators	Anterior cutaneous branch of 3rd ICN	Nerve coaptation was significantly associated with lower monofilament values in all areas of the reconstructed breast (adjusted difference, −1.2; *p* < 0.001); DIEP (+) sensory recovery was superior and started earlier postoperatively, with mean monofilament value decreasing by 0.083 per month in DIEP (+) and 0.012 in DIEP (−).
				DIEP (+) (*n* = 36)	15 months (IQR 11–17)					
Bijkerk [31]	2020	Breast Cancer Res Treat.	Partially Retro & Pros	DIEP (−/+) (*n* = 12) LTP (−/+) (*n* = 3)	18.9 ± 5.2 months	End-to-end; 9-0 nylon; 2 stitches; epineural microsutures; fibrin sealant	SWM	ICN 10–12 in DIEP flaps; LFCN/ACFN in LTP flaps	Anterior cutaneous branch of the 2nd or 3rd ICN	DIEP/LTP (+) had improved sensory recovery in all flap skin areas (*p* < 0.001), with protective sensation maintained compared to DIEP/LTP (−); Longer follow-up periods correlate with lower monofilament values in both innervated and non-innervated breasts
Cornelissen [32]	2018	Breast Cancer Res Treat	Retro	DIEP (−) (*n* = 14)	14.8 ± 4.3 months	End-to-end; 10-0 nylon; 2 stitches; epineural, microscope	SWM	Sensory nerve of DIEP flap	2nd or 3rd ICN	DIEP (+) had better pressure sensation (4.35) than DIEP (−) (5.30) (*p* < 0.01); BREAST-Q score for the domain physical well-being of the chest was 77.89 ± 18.89 on average in patients with nerve coaptation and 66.21 ± 18.26 in patients without nerve coaptation (*p* = 0.09)
				DIEP (+) (*n* = 18)	16.1 ± 3.2 months					
Blondeel [33]	1999	Br J Plast Surg.	Pros	Control (*n* = 43)	—	End-to-end; 10-0 nylon; 2 simple stitches (Ethilon, Somerville, NJ, USA)	Pressure (SWM) Vibration (tuning forks, 30 and 256 Hz) Temp. (metal probes, 2 or 42C) SEP (0.2 ms, 2 Hz)	Pure sensory branch of 10th or 11th ICN	Anterior ramus of lateral branch of 4th ICN > posterior ramus of 4th > 3rd or 5th ICN	DIEP (+) flaps had statistically significant lower pressure thresholds, with more segments reacting to cold, warm, and vibratory stimuli compared to DIEP/TRAM (−); Patient satisfaction was highest in DIEP (+) with 30% of patients showing return of erogenous sensation
				TRAM (−) (*n* = 26)	19.9 months (12–39)					
				DIEP (−) (*n* = 12)	19.6 months (12–37.8)					
				DIEP (+) (*n* = 23)	21.4 months (12.8–40)					
Beugels [34]	2021	Plast Reconstr Surg.	Pros	LTP (−) (*n* = 18)	Median: 15 months (IQR 11–25)	End-to-end; 9-0 nylon; microsurgical	SWM	Branch of lateral femoral cutaneous	Anterior cutaneous branch of 3rd ICN	LTP (+) flaps had sensory recovery that was significantly better than LTP (−), reaching diminished light touch in native skin (monofilament values 3.22–3.61) and diminished protective sensation in flap skin (3.84–4.31); Lower mean monofilament values observed for each area of LTP (+) and LTP (−) compared to DIEP flaps in another study.
				LTP (+) (*n* = 24)	Median: 17 months (IQR 10–19)					
Mori [35]	2011	Microsurgery	Retro	pTRAM (*n* = 28)	—	End-to-end	SWM; Pain (algesiometer) Temp. (metal probe, 10 or 50C)	Anterior cutaneous of 10th–11th ICN	Lateral cutaneous branch of 4th ICN	Innervated flaps demonstrated significantly greater sensitivity to touch and pain than non-innervated flaps (*p* < 0.05); TM with innervated flap showed better sensory recovery than NSM or SSM, regardless of whether sensory reconstruction is performed
				VRAM (*n* = 5)	—					
				TM (−) (*n* = 5)	31.6 months (14–57)					
				TM (+) (*n* = 5)	14.8 months (12–19)					
				NSM (−) (*n* = 8)	13.8 months (12–17)					
				NSM (+) (*n* = 6)	13.0 months (12–18)					
				SSM (−) (*n* = 5)	14.2 months (12–18)					
				SSM (+) (*n* = 4)	13.8 months (12–18)					
Puonti [36]	2011	J Plast Reconstr Aesthet Surg	Retro	TRAM (−) (*n* = 20)	54 months (27–77)	End-to-end or end-to-side; 9-0 nylon; epineural window for side-to-side	SWM; Sharp/blunt Vibration(32 and 256 Hz tuning fork) 2-point discrimination Temp./pain (Thermotest device)	10th–12th ICN	Thoracic intercostal, thoracodorsal, or intercostobrachial (one case used internal mammary vessels)	TRAM (+) showed significantly better sensory recovery than TRAM (−), with median (quartiles) of total sensory scores in the operated breasts was 12.9 (9.5–19.2) in TRAM (+) and 8.1 (3.5–10.7) in TRAM (−); Operative time in TRAM (+) was 15 min longer on average
				TRAM (+) (*n* = 20)	32 months (23–43)					
Temple [37]	2006	Plast Reconstr Surg.	Pros	TRAM (−) (*n* = 12)	16 months	End-to-end; 9-0 nylon; 2–3 simple stitches; epineural	Pressure (SWM, WEST device) 2-PD (Disk-Criminator) Temp. (tubes with water, 16 or 43C)	T10 nerve followed to lateral edge of rectus sheath; internal mammary or subscapular system used for anastomosis	Lateral cutaneous branch of 4th ICN	TRAM (+) had significantly improved postoperative pressure threshold and temperature discrimination compared to TRAM (−); TRAM (+) regained sensation throughout while TRAM (−) had increasing sensibility from the center toward the periphery.
				TRAM (+) (*n* = 15)	15 months					
Yap [38]	2005	Plast Reconstr Surg.	Pros	TRAM (−) (*n* = 7)	40 months (31–46)	End-to-end; interrupted sutures; 8-0 Ethilon nylon; epineural (microscope)	Pressure (SWM, sensory topogram) Temp. (metal probe, 5 or 60 C)	Single thoracoabdominal nerve	Lateral cutaneous branch of 4th or 5th ICN	TRAM (+) flap skin had better sensitivity to fine touch and temperature differentiation than flap skin in TRAM (−), with sensory recovery beginning earlier at 4–6 months post-op versus 12–14 months
				TRAM (+) (*n* = 7)	39 months (35–46)					
Yano [39]	1998	Plast Reconstr Surg.	Pros	pTRAM (−) (*n* = 16)	24.1 months (11–41)	End-to-end; 10-0 nylon; epineural & perineural (microscope)	SWM; Pain (algesiometer) Temp. (thermoesthesiometer, 0 or 60C)	11th ICN > 10th ICN or subcostal	Lateral cutaneous branch of 4th ICN > 3rd or 5th > anterior cutaneous branch	TRAM (+) showed return of pressure, pain, and temperature starting at 6 months with rapid recovery from the center; TRAM (−) had slow recovery (>2 years) and was poorest in center of the flap
				pTRAM (+) (*n* = 15)	14.0 months (4–24)					
Spiegel [40]	2013	Plast Reconstr Surg Glob	Retro	DIEP (−); DIEP (+); DIEP (+cnd), polyglycolic acid Total *n* = 35	182.3 ± 115.5 weeks; 119.3 ± 57.5 weeks; 88.1 ± 36.2 weeks	9-0 nylon for direct coaptation; 8-0 nylon for 40 mm NeuroTube conduit	PSSD	Pure sensory branch of T11 or T12	Anterior cutaneous branch of the 3rd ICN	DIEP flap neurotization (+ and +cnd) both significantly improved sensory recovery compared to native mastectomy skin, with nerve conduit yielding better sensory recovery and lower pressure threshold at superior/lateral/center areas than direct coaptation.
Yano [41]	2002	Plast Reconstr Surg.	Retro	LD (−) (*n* = 10)	26.9 months (15–49)	End-to-end; 10-0 nylon; epineural & perineural (microscope)	Pressure (SWM) Pain (algesiometer) Temp. (thermoesthesiometer, 0 or 60C)	Lateral cutaneous branch of dorsal divisions of 7th thoracic >6th or 8th	Lateral cutaneous branch of 4th ICN > 3rd or 5th > anterior cutaneous branch	LD (+) showed return of pressure, pain, and temperature beginning at 6 months with gradual approach to normal at 1 year; LD (−) showed slower recovery (>1 year) and was poorest in the center of the flap
				LD (+) (*n* = 4)	19.3 months (14–29)					
Blondeel [42]	1999	Br J Plast Surg.	Pros	SGAP (−) (*n* = 14) SGAP (+) (*n* = 2)	11.1 months (3.1–21.6)	End-to-end;	SWM; sensory evoked potentials for 2 flaps	Dorsal branches of 2nd or 3rd lumbar segmental (nervi clunium superiores)	Anterior ramus of lateral branch of 4th ICN	SGAP (+) showed signs of returning superficial and erogenous sensation 5 and 7 months post-operatively; inconsistent anatomy at donor site but indications are the same as myocutaneous gluteal flaps
Djohan [43]	2023	Plastic and Reconstructive Surgery	Retro	DIEP (+; allo & cnd) (*n* = 42 breasts) MS-TRAM (+allo & cnd) (*n* = 10 breasts) fTRAM (+allo & cnd) (*n* = 8 breasts) DIEP (−) (*n* = 10 breasts) MS-TRAM (−) (*n* = 5 breasts) fTRAM (−) (*n* = 3 breasts)	Nonneurotized = 14.94 ± 6.62; Neurotized = 13.48 ± 7.70	End-to-end microsurgical coaptation with 70 mm Avance allograft with AxoGard conduit; 9-0 nylon suture (1 per side), epineural	PSSD	3rd or 4th anterior ICN	Cutaneous sensory branches of T10–T12 ICN on deep flap surface	At >12 months, dynamic sensation was significantly better in neurotized vs. non-neurotized breasts (38 ± 21.7 vs. 56.2 ± 20.8 g/mm^2^, *p* = 0.014); static sensation trended better but was not significant; lower BMI (*p* = 0.012), prophylactic surgery (*p* = 0.004), nipple-sparing mastectomy (*p* = 0.006), and no radiation (*p* = 0.020) or hormonal therapy (*p* = 0.008) predicted better outcomes.
Momeni [44]	2021	PRS Global Open	Pros	TRAM or DIEP(+allo) (*n* = 15) TRAM or DIEP(−) (*n* = 14)	≥12 months	End-to-end with processed human nerve allograft; 9-0 nylon, microsurgery	SWM	11th or 12th ICN	Anterior cutaneous branch of the 3rd ICN	Flap neurotization resulted in a greater return of protective sensation; Neurotized breasts showed a greater likelihood for return of sensation in 8 of 9 examined zones; 55% of neurotized breasts had protective sensation in ≥5 zones, compared to 7% in non-neurotized breasts (*p* < 0.01); 64% of non-neurotized flaps had no return of protective sensation versus 27% of neurotized flaps (*p* = 0.04)
Tevlin [45]	2021	J of Plastic, Recon, & Aesth Surg	Retro	Autologous flap (type not specified) (+allo or auto) (*n* = 12 and *n* = 2) Autologous flap (type not specified) (−) (*n* = 20)	Minimum 8 months; median 36 months in control group	End-to-end to a cadaveric nerve graft or autologous graft; epineurally sutured (7-0 prolene); loupe magnification	SWM	Lateral ICN (T3–T5)	Base of NAC or dermis	Neurotized breasts had significantly better whole-breast sensation (mean 4.8 ± 1.5 vs. 5.4 ± 1.0, *p* = 0.0001), improved areolar sensation (*p* = 0.0001), and preservation of nipple sensation compared to baseline (*p* = 0.096); control group showed significant loss of nipple sensation postoperatively (*p* = 0.0001)
Carrau [46]	2022	Annals of Breast Surg	Retro	N = 52 total, Number per group not detailed DIEP (+allo) DIEP (−)	Minimum 6 months, with assessments at 3, 6, and 12 months	Graft-bridged end-to-end; two interrupted 9-0 nylon, epineurally sutured under loupe magnification	SWM	T10–T12 intercostal sensory branches within the flap	Anterior cutaneous branch of the 3rd or 4th ICN	At 12 months, 93% of neurotized vs. 87% of non-neurotized flaps had regained sensation; both groups recovered sensation in 2/9 zones on average; neurotized flaps had slightly better monofilament thresholds (5.18 g vs. 5.43 g), but not clinically significant; sensory return occurred earlier and more frequently in neurotized flaps; Slightly higher rate and area of sensation recovery in neurotized flaps but difference narrowed at 12 months; literature review supports earlier, more complete, and erogenous sensory return with neurotization
Zhang [47]	2025	Annals of Plastic Surgery	Pros	DIEP (+allo) (*n* = 112) Implant (−) with TE (*n* = 82)	Sensory data collected at 6 m, 12 m, 24 m, and 24+ months	Single neurorrhaphy with 70 mm Avance nerve allograft (Axogen); microsurgical	PSSD	Sensory nerve per Spiegel et al. (likely 10th–12th ICN, as per prior studies)	Anterior 3rd ICN	In the autologous cohort, NAC sensitivity significantly correlated with higher psychosocial (β = −0.20, *p* = 0.01) and sexual wellbeing (β = −0.26, *p* = 0.04); overall breast sensitivity correlated with satisfaction with breasts on univariate analysis, but not multivariate; no breast region correlated with physical wellbeing scores. In the alloplastic cohort, only NAC sensitivity correlated with sexual wellbeing (β = −0.10, *p* = 0.002); no other domains showed significant associations.
Black [48]	2024	Annals of Plastic Surgery	Pros	DIEP (+allo) (*n* = 106) 2-stage alloplastic w TE (−) (*n* = 86)	1072 ± 392.6 days (~3 years); 1875 ± 1029.3 days (~5 years)	End-to-end with 70 mm Avance allograft (Axogen); microsurgical	PSSD	Sensory branch of T10–Th12	Anterior cutaneous branch of the 3rd ICN	At 1 year, the DIEP cohort showed significantly better sensation than the alloplastic group in 5 of 9 regions (including NAC and inner regions); at 4 years, this expanded to 7 of 9 regions; sensation improved most in the NAC (28.9 g/mm^2^) and outer lateral breast (30.4 g/mm^2^) in the DIEP group; alloplastic cohort had greater improvement at NAC than other regions, but lagged behind DIEP overall; average sensation thresholds at 4 years were 14.3 g/mm^2^ better in autologous vs. alloplastic reconstructions (*p* < 0.05).
Huang [49]	2022	Annals of Plastic Surgery	Pros	DIEP (+allo) (*n* = 41) TE (−) (*n* = 46)	Minimum 8 months; median 36 months in control group	End-to-end coaptation with Avance allograft (Axogen); microsurgical	PSSD (AcroVal, AxoGen)	Sensory branch of T10–T12	Anterior cutaneous branch of the 3rd ICN	In DIEP patients, sensation in outer regions returned to baseline by 18 months and nearly all regions by 3 years, except inner inferior (*p* = 0.016); TE patients had significantly worse sensation than baseline in all regions at 5 years (*p* < 0.05); BREAST-Q scores trended higher in DIEP patients for all domains but differences were not statistically significant (*p* > 0.05).
Zhang [50]	2025	Ann Plast Surg	Retro	DIEP (+coap) (*n* = 11)	92.67 months [60.35, 112.52]	Direct end-to-end coap; Coap with allo using 70 mm nerve	PSSD (AcroVal, AxoGen)	T10–12	Anterior cutaneous branch of 3rd ICN	Overall breast cutaneous sensitivity measurement was 64.58 g/mm^2^ [40.06, 78.99] in the direct coaptation group and 78.28 g/mm^2^ [40.60, 82.06] in the nerve allograft group, with no significant differences overall (*p* = 0.680) or at any specific breast area. BREAST-Q surveys were comparable across all scales.
				DIEP (+allo) (*n* = 19)	84.79 months [65.05, 88.21]					
Lu Wang [51]	2023	Annals of Plastic Surgery	Pros	Nipple-sparing & buried DIEP (+allo) (*n* = 60) Skin-sparing & nonburied DIEP (+allo) (*n* = 10)	Up to 24 months; sensory testing at baseline, 6 mo, and 24 mo	End-to-end with 70 mm Avance nerve graft; microsurgical	PSSD (AcroVal, AxoGen)	Sensory branch of T10–T12 thoracoabdominal nerves	Anterior cutaneous branch of 3rd ICN	At 6 months, buried flap patients had significantly worse inner breast region sensation compared to baseline (10.19 vs. 70.83 g/mm^2^, *p* < 0.001), whereas nonburied flap patients showed no significant difference from baseline (27.54 vs. 57.24 g/mm^2^, *p* = 0.236); by 24 months, both groups returned to baseline sensitivity (*p* > 0.05); baseline sensitivity was significantly higher in the buried group preoperatively (*p* < 0.01 across all regions).
Lu Wang [52]	2023	Annals of Plastic Surgery	Pros	Immediate DIEP (+allo) (*n* = 65) Delayed-Immediate DIEP after tissue expander (+allo) (*n* = 26)	Up to 24 months post-mastectomy	End-to-end with 70 mm Avance nerve graft; microsurgical	PSSD (AcroVal, AxoGen)	Sensory branch of T10–T12 thoracoabdominal nerves	Anterior cutaneous branch of 3rd ICN	At 18 months post-mastectomy, both cohorts showed similar sensitivity in all breast regions (*p* > 0.05); by 24 months, sensitivity returned to baseline in all regions except the inner inferior quadrant (*p* = 0.016); BREAST-Q scores were not significantly different between cohorts at 18 or 24 months (*p* > 0.05), but psychosocial (*p* = 0.18) and sexual well-being (*p* = 0.08) trended higher in delayed-immediate patients.
Puonti [53]	2017	Clin Breast Cancer.	Partially retro & pros	ms-TRAM (+cnd) (*n* = 29)	29.9 ± 5.8 months (24–43)	9-0 nylon; perineural; 2–3 sutures (end-to-end); or 3–4 sutures (end-to-side); NeuraGen 3 mm diam. Conduit	Pressure (SWM) Temperature (thermostat) Vibration (32 and 256 Hz tuning fork) Sharp/blunt (pin) 2-PD	10th–12th ICN	Medial: 3rd or 4th ICN Lateral: costobrachial, thoracodorsal, branches of 4th or 5th ICN in axillary	Dual neurorrhaphy had better median total sensory scores after 2-year follow-up, including tactile, cool detection, and nipple sensation compared to single neurorrhaphy (*p* = 0.037); no differences in operation times between dual and single neurorrhaphy (*p* = 0.0328); Dual neurorrhaphy may restore 60% of healthy breast sensation compared to 45% in single neurorrhaphy Questionnaire: slightly higher patient satisfaction in dual neurorrhaphy (median 9.5, IQR 8.6–10) vs. single neurorrhaphy (median 9.0, IQR 8–9)
				ms-TRAM (++cnd) (*n* = 41)	25.7 ± 2.4 months (23–36)					
Puonti [54]	2017	Clin Breast Cancer.	Partially retro & pros	Control (*n* = 56) ms-TRAM (−) (*n* = 20) ms-TRAM (++cnd) (*n* = 38)	1–2+ years 54 months (27–77); 1–2+ years	End-to-end or end-to-side with 9-0 nylon; perineural; 2 sutures; NeuraGen 3 mm diam. conduit	Pressure (SWM) Thermostat; Vibration (32 and 256 Hz tuning fork); 2-PD; Somatosensory evoked potential (SEP) Biopsy (ENFD)	10th–12th ICN	Medial: 3rd or 4th ICN Lateral: intercostobrachial	Sensory recovery in total peripheral nerve surgery can occur via collateral reinnervation from neighboring areas even if neurrorrhaphy on injured nerve is not performed; ms-TRAM (++cnd) dual neurrhaphy showed better median total sensory scores than ms-TRAM (−) when using tests that measured large fiber function (SEP, SENFD)
Temple [55]	2009	Plast Reconstr Surg.	Pros	TRAM (−) (*n* = 8)	48 months	End-to-end; 9-0 nylon; 2–3 simple stitches; epineural	-	T10 nerve followed to lateral edge of rectus sheath	Lateral cutaneous branch of 4th ICN	Patient rated higher QoL improvements in TRAM (+) compared to TRAM (−) in the majority of domains, which included physical function, body image, and emotional well-being
				TRAM (+) (*n* = 10)	-					

TRAM = transverse rectus abdominis myocutaneous; VRAM = vertical rectus abdominis myocutaneous; SGAP/IGAP = superior/inferior gluteal artery perforator; DIEP = deep inferior epigastric perforator; LD = latissimus dorsi; LTP = lateral thigh perforator; TM = total mastectomy; NSM = nipple-sparing mastectomy; SSM = skin-sparing mastectomy; ms = muscle sparing; − = no neurotization; + = single neurorrhaphy; ++ = dual neurorrhaphy; cnd = conduit; allo = allograft.

**Table 2:** Summary of studies evaluating sensory restoration of breast reconstruction with various neurotization techniques in implant-based reconstruction.

Author	Year	Journal	Design	Recon Type	Follow Up Mean ± SD [Range]	Method	Sensory Testing	Donor Nerve	Recipient Nerve	Key Results
Zhang [56]	2024	J Reconstr Microsurg.	Retro	Direct-to-implant (+allo) (*n* = 33) Tissue expander (+allo) (*n* = 23)	3, 6, and 12 months	End-to-end coap with allo; 9-0 and 8-0 nylon	SWM; 5 per breast	Lateral Th3-5 ICN	Undersurface of the NAC	At 12 months, significant improvement in monofilament thresholds across all NAC and breast skin regions compared to earlier timepoints (*p* < 0.001); average total nerve length was 12.3 cm from nerve origin to NAC; no chronic pain, neuroma, or dysesthesia
Peled [57]	2023	Plast Reconstr Surg.	Pros	Direct-to-implant (+allo) (*n* = 47)	9.2 months (6–14 months)	End-to-end coap with allo	PSSD; 5 per breast	Lateral Th3-5 ICN	Subareolar nerve branches	At 12 months, 75% of one-point moving (1 PM) and 38–75% of one-point static (1 PS) thresholds across all breast and NAC areas tested were in the “excellent” range (<20 g/mm^2^); no chronic pain
Djohan [58]	2020	Plast Reconstr Surg.	Pros	Direct-to-implant (+allo) (*n* = 11 breasts) Tissue expander (+allo) (*n* = 4 breasts)	1st follow-up: 4.2 ± 2.3 months 2nd follow-up: 10.6 ± 3.6 months	Coap with allo; 9-0 nylon	PSSD; 8 per breast	Lateral Th4 ICN	Processed nerve allo	Neurotized breasts had better thresholds in 6/8 areas compared to non-neurotized breasts; Sensory recovery best at superior and upper-inner quadrant breast areas; All areas had better sensation at 2nd follow-up
Peled [59]	2019	Plast Reconstr Surg Glob	Pros	Direct-to-implant (+; allo) (*n* = 16)	3.6+ months	Coap with allo; 8-0 or 9-0 nylon	Gross, light touch (2-PD)	Th4 or Th5 lateral ICN	1–2 mm diameter Avance nerve allo	NAC 2-point discrimination was preserved in 20 breasts (87%), worse in 2 breasts (9%), and improved in 1 breast (4%); 67% reported similar overall re-op/post-op breast and NAC sensation; no neuromas or dyesthesias
Shyu [60]	2025	Int Jour of Surg	Pros	DIEP (+autologous nerve graft) (*n* = 54) PAP (+autologous nerve graft) (*n* = 6) Implant (+autologous nerve graft) (*n* = 7) DIEP (−) (*n* = 27) PAP (−) (*n* = 3) Implant (−) (*n* = 35)	1.3 ± 0.5 years	End-to-end autologous graft from ICN to nipple base; 9-0 nylon	SWM	Main branch of Th3-Th5 ICN (typically 4th)	Lateral cutaneous branch to nipple base	Better nipple sensation in innervated group compared to non-innervated groups (2.6 ± 1.2 vs. 1.9 ± 1.0, *p* = 0.002); monofilament values were significantly correlated with patient-reported psychosocial well-being (*p* = 0.033), nipple (*p* = 0.008), and breast sensation (*p* = 0.009)
Chang [61]	2024	Brit Jour Surg	Retro case-control	DIEP (+autologous nerve graft) (*n* = 53) Implant (+autologous nerve graft) (*n* = 3) DIEP (−) (*n* = 10) Implant (−) (*n* = 5) PAP (−) (*n* = 1)	Up to 24 months	End-to-end coap; autologous graft elongation (20–25 cm) from ICN to NAC	SWM + MRC scale; 5 per breast	Main branch of Th3-Th5 ICN (preferably 4th)	Base of the nipple; dermis for future NAC (in non-preserving cases)	Positive control breasts had mean monofilament values of 0.07 g and 179.13 g for the non-neurotized negative control group; Monofilament results for neurotized breasts improved from 138.2 g at 0–6 months to 0.37 g at 19–24 months (*p* < 0.001); sensation recovered faster in delayed vs. immediate reconstructions; no neuromatous pain reported
Juan [62]	2024	Front in Onc	RCT	Subpectoral prosthetic titanized polypropylene mesh (TiLOOP^®^, pfm medical, Cologne, Germany) Implant (+) (*n* = 50) Implant (−) (*n* = 53)	6 months	End-to-end coap of ICN to NAC tissue	SWM; 9 per breast	Lateral cutaneous branches of Th2–Th4 ICN	Subareolar dermis of NAC	At 6 months, neurotized patients had significantly better nipple (*p* < 0.001), areola (*p* = 0.06), and breast skin sensation (*p* = 0.01) vs. control; operative time increased by ~20 min (*p* < 0.001) with no increase in complications, blood loss, or drainage volume.

SWM = Semmes-Weinstein monofilament; 2-PD = 2-point discrimination; PSSD = pressure specified sensory device; NAC = nipple areolar complex; ICN = intercostal nerve; PAP = profunda artery perforator; DIEP = deep inferior epigastric perforator; − = no neurotization; + = single neurorrhaphy; allo = allograft.

While the DIEP flap was the most frequently studied, others also examined LTP, free TRAM, muscle sparing-TRAM (ms-TRAM), pedicled TRAM (pTRAM), LD, thigh-based, and SGAP flaps. Eight studies evaluated direct coaptation in TRAM flaps, with Slezak et al. being the first to report earlier recovery of vibratory sensation and localized touch in sensate reconstruction [8,25,26,35,36,37,38,39]. Puonti et al. found that any available nerve repair resulted in improved sensory recovery compared to non-innervated reconstruction, with the best sensation found for an end-to-side anastomosis between the thoracodorsal nerve and the 12th thoracoabdominal nerve (T12 ICN) [36]. Temple et al., Yap et al., and Isenberg et al. found similar results showing that innervated TRAM flaps exhibited more uniform and earlier sensory return (4–6 months) compared to their controls (12–14 months), suggesting both peripheral nerve ingrowth and flap innervation from the center [25,37,38]. This pattern of recovery was further supported in a prior study by Yano et al., which indicated that innervated TRAM flaps had rapid recovery from the center starting at 6 months after surgery, whereas non-innervated TRAM flaps gradually began recovery at 10 months with poorest sensation in the center of the flap [39]. The neurotization component for direct coaptation in DIEP and TRAM flaps were noted to range from 10 to 35 min, respectively, indicating that minimal surgical time is added to produce significant sensory results [37,40].

Fewer studies have looked at alternative forms of autologous reconstruction, such as LD, LTP, and SGAP flaps. Beugels et al. and Bijkerk et al. found that coaptation with LTP flaps significantly improved tactile thresholds over time in all areas of the reconstructed breast, except for the lower medial and lower lateral part of the native skin [31,34]. Isenberg et al. found that innervated TRAM flaps showed greater sensory recovery compared to innervated LD flaps, while Yano et al. found that innervated LD flaps had a faster recovery than LD flaps that were not neurotized [25,41]. Blondeel et al. was the only study included in this review to evaluate neurotized SGAP flaps, which showed signs of recovery of superficial and erogenous sensation at 5 and 7 months post-operation (*n* = 2) [42]. Almost all studies using direct coaptation supported neurotization in breast reconstruction, with the lowest “success” rates ranging from 37.5% for SGAP flaps to 82.8% for DIEP flaps [29,42].

### 3.2. Autologous Reconstruction: Coaptation with Nerve Allograft or Conduit

Eleven studies from 2021 to 2025, incorporating 901 patients, investigated interpositional nerve grafting in autologous breast reconstruction. These studies used either processed nerve allografts (e.g., Axogen Avance) or autologous donor nerves (e.g., main intercostal nerve) to bridge gaps between donor and recipient nerves. The recent shift toward nerve grafting reflects its utility in cases where direct coaptation is precluded by nerve length discrepancy, suboptimal flap orientation, or recipient nerve inaccessibility.

Four studies directly compared nerve allografting to non-neurotized flaps [43,44,45,46]. Djohan et al. examined 78 flaps across DIEP, TRAM, and PAP reconstructions, reporting significantly higher static and dynamic sensation in neurotized breasts (with allograft and conduit) after 12 months, but did not show significance for static sensation [43]. Similarly, Momeni et al. directly compared nerve-grafted versus non-neurotized autologous reconstructions using a standardized intercostal-to-flap nerve allograft technique, showing 64% of non-neurotized flaps had no return of protective sensation versus 27% of neurotized flaps (*p* = 0.04) [44]. Tevlin et al. utilized a novel lateral intercostal-to-nipple areolar complex (NAC) allograft tunneling procedure and observed consistently better monofilament thresholds in the allograft group in whole breast sensation (*p* = 0.0001) and areolar sensation (*p* = 0.0001), compared to matched non-neurotized controls [45]. Carrau et al. reported 93% of DIEP flaps with both an allograft and conduit demonstrated measurable recovery on monofilament testing by 12 months, compared to 87% in non-neurotized flaps [46]. Though not powered for significance, the sensory testing at 3- and 6-month timepoints supported earlier sensory return.

One study compared direct coaptation to nerve allografting. Zhang et al. compared 18 DIEP flaps neurotized with direct end-to-end coaptation and 19 with decellularized nerve allograft bridging [24]. Both cohorts recovered sensation in all breast quadrants and the NAC by 12 months. There was no significant difference in mean breast sensitivity between direct (64.58 g/mm^2^) and allograft (78.28 g/mm^2^) groups (*p* = 0.680). Furthermore, there was no statistical difference in regional sensation or BREAST-Q scores, suggesting grafting as a viable alternative when direct alignment is not feasible.

Three studies evaluated DIEP nerve allografting compared to various non-neurotized comparators, including tissue expander reconstructions and delayed autologous flaps [47,48,49]. Zhang et al. compared nerve allograft DIEP flaps to a non-neurotized 2-stage alloplastic reconstruction group [47]. Neurotized breasts showed statistically significant improved sensitivity in the outer region of the breast and the NAC at 12–24 months. However, after 24 months, both groups showed comparable sensitivity in all regions of the breast. Black et al. found that an allografted DIEP flap cohort showed significantly better sensation in 5 of 9 regions (including NAC and inner regions) relative to the non-neurotized 2-stage alloplastic reconstruction group [48]. At 4 years, this expanded to 7 of 9 regions. Huang et al. showed cutaneous thresholds in nerve allograft DIEP flaps returned to preoperative baseline except the inner inferior region at 3 years (*p* > 0.05), while TE/implant-based reconstructions continued to be significantly worse in the entire breast at 5 years (*p* < 0.05) [49].

Three studies assessed the impact of flap configuration or donor nerve choice on sensory outcomes in neurotized DIEP flaps [50,51,52]. Zhang et al. compared reconstructions using the Th11 versus Th12 ICN with allograft [50]. Use of the twelfth nerve resulted in significantly better sensitivity across the breast, particularly in the medial and central zones (*p* = 0.01). This suggests the selection of a more caudal intercostal nerve may improve neurotization outcomes. Lu Wang et al. compared buried versus non-buried DIEP flaps, with both cohorts receiving neurotization using lateral intercostal nerves and nerve allografts [51]. Buried flaps had superior sensation in the inner breast (*p* = 0.006) and NAC (*p* = 0.012) at 6 months, possibly due to closer nerve alignment and reduced graft angulation. A second study by Lu Wang et al. compared nerve allografting of immediate and staged or delayed-immediate DIEP reconstructions, both neurotized at flap inset [52]. Sensory outcomes at 18 months were comparable between groups in most regions, demonstrating equivalent sensory recovery across both methods of flap neurotization.

Three studies examined direct coaptation with a conduit, with two articles using a 3 mm diameter NeuraGen tube (Integra LifeSciences Corp., Plainsboro, NJ, USA) [40,53,54]. Puonti et al. examined single neurorrhaphy with a conduit compared to dual neurorrhaphy with a conduit in 70 ms-TRAM patients, and found that dual neurorrhaphy had better median total sensory scores (tactile, cool detection, and nipple sensation) compared to single (*p* = 0.037) and non-neurotized flaps after a 2-year follow-up [53,54].

Compared to non-neurotized flaps, reconstructions using nerve allografts exhibit earlier return and more uniform recovery of NAC and flap sensation supporting nerve allografting as an effective approach when direct neurorrhaphy is not viable. When coapting using conduits, dual neurorrhaphy also offered better sensory recovery than single neurorrhaphy and non-neurotized flaps, with improved sensory recovery in the superior/lateral/center areas, though more robust investigation is warranted [40].

### 3.3. Implant-Based Reconstruction: Nerve Allograft or Direct Neurorrhaphy

Seven studies examined sensory outcomes for neurotized implant-based reconstructions, with four using processed nerve allografts [56,57,58,59], two using autologous nerve grafts [60,61], and one study using direct neurorrhaphy for neurotization [62]. Zhang et al. conducted a retrospective study of patients who underwent reconstruction with either direct-to-implant (DTI) or staged tissue-expander reconstruction with a nerve allograft [56]. The average length of ICN harvested was 5.3 cm, and the total nerve length with the allograft reached 12.6 cm for DTI and 12 cm for staged reconstruction. Out of 58 breasts, 54 were successfully neurotized and 4 were aborted due to insufficient length. Significant improvements were shown in monofilament thresholds across all NAC and breast skin regions at 12 months compared to 3 and 6 months, with no abnormal sensations such as chronic pain and dysesthesias over time. However, this study lacked a control group and was limited to small/medium implants due to nerve length constraints. Peled et al. conducted two studies (with a total of 110 breasts) examining the use of allografts in direct-to-implant reconstruction, with one study reporting rates of up to 90% preserved sensation to gross and light touch of the breast [59]. At 12 months post-operatively, 75% of one-point moving pressure threshold and 38–75% of one-point static threshold measurements were excellent across all breast and NAC areas tested (<20 g/mm^2^) [57]. Similar to Zhang et al., none of the patients reported chronic, post-mastectomy pain at 6 months. Djohan et al. found sensory recovery to be the best at the superior and upper-inner quadrant breast and NAC areas for static and dynamic tests, respectively, in implant-based reconstruction [58]. However, almost all studies lacked a non-neurotized control group, had varying methods for sensory measurements, and were limited by small sample sizes.

Shyu et al. and Chang et al. reported on the novel technique of autologous nerve grafts for neurotization by utilizing the main branch of the intercostal nerve as a graft to elongate the lateral cutaneous branch of the 4th intercostal nerve [60,61]. The graft was then coapted to the base or dermis of the nipple, with an average nerve length of 20–25 cm from the proximal nerve to the distal graft tip. Chang et al. reported an additional 1.5–2 h of time needed in surgery for graft preparation, and successfully completed neurotization in 55 out of 56 planned cases, with one initial failure later revised [61]. Despite having a longer follow-up after surgery, the non-neurotization group had poor results in the mean monofilament test for the nipples (179.13 g) compared to neurotized breasts (35.61 g) and control contralateral breasts (0.07 g). Although complications in this study included pleural ruptures that occurred due to its proximity to the intercostal nerve donor, the compromise to respiratory function is limited.

Juan et al. examined the safety and efficacy of direct end-to-end intercostal nerve anastomosis for subpectoral prosthetic breast reconstruction using the T2–4 ICNs and the subareolar dermis of the NAC [62]. There was a significant return of local sensation in the NAC for innervated breasts as early as 3 months. Compared to the control group that received implant-based reconstruction without intercostal nerve anastomosis, the operative time for the treatment group significantly increased by 20 min (*p* < 0.001). Although there were minimal complications, the authors noted that the recovery in both the control group and the treatment group was not as good as in previous studies, likely due to the thinner layer of subcutaneous fat used in the operation.

### 3.4. Quality of Life

Patient-reported outcomes (PROs) have become increasingly central to evaluating breast surgery. This is especially important for the evaluation of neurotization techniques, which are often performed with the goal of improving long-term satisfaction. Overall, 40% (*n* = 16) of studies included assessed quality of life (QoL), with the most utilized tool being the BREAST-Q survey, a validated tool that encompasses multiple domains including psychosocial, sexual, and physical well-being. Other QoL assessment tools included the EORTC QLQ-C30, the Patient Satisfaction Questionnaire, the Medical Outcomes Study 36-Item Short Form Health Survey, the Body Image after Breast Cancer Questionnaire, the Functional Assessment of Cancer Therapy-Breast quality-of-life instrument (FACT-B), and custom study-specific instruments.

In a 2025 study, Zhang et al. found no significant differences in BREAST-Q scores between patients undergoing DIEP flap reconstruction with either direct end-to-end nerve coaptation or nerve allograft [24]. A separate study by the same group compared DIEP flaps neurotized with T11 versus T12 donor nerves and again found no significant difference; however, patients in the T12 group had higher QoL scores with a trend toward significance and may be considered as the preferred choice for donor nerves based on these findings [50]. Similarly, Puonti et al. found no significant QoL difference between patients undergoing single versus dual neurorrhaphy techniques, as measured by the Patient Satisfaction Questionnaire [53]. Further supporting the potential versatility of neurotization, Lu et al. found no significant difference in BREAST-Q scores when comparing immediate versus delayed DIEP reconstruction with nerve allograft [52].

When comparing neurotized versus non-neurotized reconstruction, several studies report similar or modestly improved quality of life in those who had neurotized breast reconstructions. For example, a study by Cornelissen et al. found no significant difference in the BREAST-Q physical well-being domain between patients who underwent DIEP flap reconstruction with direct end-to-end nerve coaptation and those without neurotization; although, this pilot study demonstrated higher scores in the neurotization group with a trend toward significance (*p* = 0.09) [32]. Puonti et al. demonstrated increased sensory recovery after nerve anastomosis in TRAM flaps; however, quality of life measures remained similar between neurotization and control cohorts [36]. Huang et al. reported comparable BREAST-Q scores between patients undergoing DIEP flap reconstruction with allograft neurotization and those who received tissue expander placement [49].

In contrast, some studies suggest more meaningful improvements in QoL associated with neurotization. Juan et al. found significantly higher scores in emotional, physical, social function, and pain domains (EORTC QLQ-C30) in patients who underwent immediate subpectoral implant-based reconstruction with direct end-to-end nerve coaptation [62]. Similarly, Temple et al. reported significantly improved QoL across three validated instruments: (1) SF-36, (2) Body Image after Breast Cancer Questionnaire, and (3) FACT-B in patients who underwent neurotized TRAM flap reconstruction compared to non-neurotized controls [55].

In the assessment of breast sensation, the NAC is often considered separately due to its key role in erogenous sensation and impact on psychosexual health. In a 2025 study, Shyu et al. demonstrated significantly higher QoL across the BREAST-Q psychosocial, sexual, and provider satisfaction domains in patients who underwent breast neurotization with nerve autograft following nipple-sparing mastectomy [60]. These patients also reported greater nipple sensation, as measured by a study-specific questionnaire. Additionally, the study demonstrated that improved sensation, as assessed by monofilament testing, was significantly associated with better quality of life outcomes, including subjective nipple and breast sensation and the BREAST-Q psychosocial domain. Likewise, Zhang et al. found that increased NAC sensation was associated with significant increases in BREAST-Q sexual and psychosocial scores following neurotized DIEP reconstruction [47]. Further evidence comes from Blondeel et al., who found that patient satisfaction and return of erogenous sensation were highest in neurotized DIEP flaps compared to non-neurotized DIEP/TRAM flaps [33]. However, not all studies support these findings. In a study by Magarakis et al., only one patient who underwent neurotized DIEP flap reconstruction “strongly agreed” that erogenous sensation was preserved postoperatively [27]. Additionally, Isenberg et al. reported that no patients in their cohort regained erogenous sensation following TRAM reconstruction with neurotization, as assessed using a custom survey tool [26].

## 4. Discussion

Over the past two decades, the recent literature consistently supports the efficacy of neurotization in improving sensory outcomes after breast reconstruction. Across both autologous and implant-based approaches, innervated breasts demonstrated an earlier and stronger return of sensation, particularly in central/medial breast zones and the NAC. This systematic review identified 40 studies: 20 of which included direct coaptation in autologous reconstruction, 16 studies which mentioned direct coaptation with allograft, autograft, or conduit, and seven studies that focused on implant-based reconstruction with attempted neurotization.

Autologous reconstruction, particularly DIEP and TRAM flaps, has been the most extensively studied, with direct nerve coaptation being the main technique used. Innervated flaps achieve robust pressure, temperature, and erogenous sensation, with success rates ranging from 83% to 100%. Certain studies defined success as a significant difference in monofilament scores, whereas others determined it as a measurable threshold improvement or subjective patient-reported responses [56,58,60]. This aligns with an earlier review that reported pooled success rates of 90.6% for nerve coaptation studies, indicating the feasibility of incorporating neurotization as a part of breast reconstruction [20]. Nerve allografting has emerged as a viable alternative when direct coaptation is not feasible due to length discrepancies or flap orientation, and studies have shown that similar sensory outcomes are achieved. Both direct coaptation and nerve allografting perform better than non-neurotized controls in terms of earlier and more uniform sensory recovery in the outer region of the breast and NAC, with higher dynamic sensation after 12 months [28,43,47]. Dual neurorrhaphy and the use of conduits have shown further improvements in sensory scores relative to single neurorrhaphy in ms-TRAM flaps, with very little differences in operation times and slightly higher patient satisfaction [53,54]. Shyu et al., Chang et al., and Tevlin et al. recently explored the use of autologous nerve grafts for elongation of the intercostal nerve to the NAC, improving pressure sensation and avoiding extra donor morbidity sites or neuromatous pain [45,60,61]. This shows a promising avenue for enhancing neurotization, particularly after NSM, with gradual sensory return occurring within the first 6 months [56,61].

In implant-based reconstruction, neurotization using processed nerve allografts or autologous nerve grafts is associated with significant improvements in sensory thresholds, although the body of evidence is smaller and may not have adequate control groups. Direct-to-implant or tissue expanders were typically placed in the submuscular plane followed by allografting, with return of sensation most pronounced in nipple and areola regions [62]. No chronic pain, neuromas, or dysthesias were reported in multiple studies, indicating decreased negative outcomes associated with cut nerve ends and post-mastectomy pain development. However, implant-based neurotization presents unique technical constraints, including limited nerve length. In these settings, autologous nerve grafts are generally reserved for longer gaps or when extended reach around the implant or expander pocket is required to bridge peripheral nerve deficiencies [63]. Alternatively, processed cadaveric grafts may also be used to bridge nerve injuries greater than 25 mm and to avoid donor-site morbidity [64]. These technical factors likely contribute to heterogeneity in reported outcomes.

While several studies demonstrate associations between improved sensation and higher psychosocial or sexual well-being scores, results are not uniformly significant and effect sizes are modest. Interestingly, higher scores in psychosocial and sexual well-being were reported in innervated DIEP flaps and implants across multiple timepoints, and even reduced symptoms like eczema and itching [60]. However, less than half of the articles assessed quality of life, and even fewer correlated objective sensory outcomes and subjective satisfaction. Despite these promising trends, several limitations and gaps persist in the current literature. Radiation therapy emerges as a potential modifier of sensory recovery. Magarakis et al. demonstrated inferior sensory outcomes in irradiated implant reconstructions compared to irradiated DIEP flaps, and Djohan et al. identified the absence of radiation as a predictor of improved dynamic sensation [27,43]. Radiation may impair axonal regeneration through fibrosis, microvascular compromise, and inflammatory changes, and thus stratified reporting by radiation status should be incorporated into future neurotization trials [65].

Interpretation of these findings must be tempered by substantial heterogeneity across the included articles. Studies differed in reconstruction type (DIEP, TRAM, or implant-based), radiation exposure, mastectomy type (skin-sparing versus nipple-sparing), timing of reconstruction (immediate versus delayed), and technique (single versus dual neurorrhaphy). Furthermore, follow-up duration ranged from 4 months to over 5 years, and sensory testing modalities varied widely. SWM testing provides a widely available but less reproducible assessment of cutaneous sensibility, whereas the PSSD offers more precise, continuous, and quantifiable measurement of pressure thresholds with greater sensitivity [66]. Two-point discrimination tests assess spatial acuity but are less reliable in reconstructed tissue due to flap thickness variability. Importantly, no standardized definition of sensory recovery exists. This lack of consensus limits cross-study comparability and calls for standardized outcome definitions that would enhance interpretability and future meta-analysis.

While current techniques continue to be optimized, the variability in sensory recovery is due in part to both patient and surgical characteristics as well as the unpredictable nature of tissue healing in the face of chemotherapy, surgery, and radiation. One solution to sensory restoration or preservation capitalizes on current knowledge of neuroprosthetics in limb amputation but applies flexible sensor technology for a fully implantable device in the setting of post-mastectomy reconstruction [67]. Furthermore, determining the impact that the sensation of the breast or reconstructed breast has on the embodiment of the reconstruction and restoration of self has yet to be determined.

## 5. Conclusions

Overall, the current body of evidence supports the benefit of neurotization in breast reconstruction for sensory recovery following mastectomies. However, heterogeneity in outcome reporting, limited randomized data, and inconsistent quality of life findings temper the strength of current conclusions. Future randomized trials using standardized sensory metrics and radiation-stratified analyses are essential to define the true clinical benefit and durability of neurotization techniques.

## Data Availability

No new data were created or analyzed in this study.

## Supplementary Materials

The following supporting information can be downloaded at: https://www.mdpi.com/article/10.3390/cancers18071052/s1, Supplemental Materials: Data research; Table S1: Risk-of-bias assessment of studies included in analysis [22,23]; Table S2: PRISMA checklist [21].
