# Supplementary material for: Sensory Outcomes and Neurotization Techniques Following Mastectomies: A Comprehensive Systematic Review
Source: Cancers (Basel). 2026 Mar 24;18(7):1052. doi: 10.3390/cancers18071052 (PMC13072177; doi:10.3390/cancers18071052)
Supplement: Supplementary file 1 [file cancers-18-01052-s001.zip › Supplemental Materials.pdf]

Supplemental Materials

Database Search:

PubMed:

("Breast Neoplasms"[MeSH Terms] OR "Mammaplasty"[MeSH Terms] OR "Post-Mastectomy" OR "Breast*") AND ("DIEP" OR "Deep Inferior Epigastric Perforator" OR "TRAM" OR "Transverse Rectus Abdominis Musculocutaneous" OR "Rectus Abdominis Myocutaneous" OR "SIEA" OR "Superficial Inferior Epigastric Artery" OR "TUG" OR "Transverse Upper Gracilis" OR "PAP" OR "Profunda Artery Perforator" OR "GAP Flap" OR "Gluteal Artery Perforator" OR "Latissimus Dorsi" OR "LD Flap" OR "Implant" OR "Breast Reconstruction"[MeSH Terms] OR "Mastectomy" OR "Tissue Expander" OR "Autologous" OR "Flap" OR "Reconstruction") AND ("Sensation"[MeSH Terms] OR "Sensory Reinnervation"[MeSH Terms] OR "Neurotization" OR "Nerve Coaptation" OR "Nerve Regeneration"[MeSH Terms] OR "Nerve Graft" OR "Nerve Transfer" OR "Nerve Repair" OR "Reinnervation"[MeSH Terms] OR "Sensory Restoration" OR "Neural Coaptation" OR "Neuroplasticity"[MeSH Terms] OR "Sensibility" OR "Sensory Recovery" OR "Nerve Preservation" OR "Nerve Conduit" OR "Nerve Allograft" OR "Resensitization" OR "Neurotis*" OR "Neurotiz*" OR "Neurorraph*" OR "Reinnervat*" OR "Sensation")

Embase:

('breast tumor'/exp OR 'breast neoplasm*' OR 'breast cancer surgery' OR 'mammaplasty'/exp OR 'post mastectomy' OR 'breast reconstruction'/exp OR 'mastectomy'/exp OR breast*:ti,ab)
AND
('deep inferior epigastric perforator' OR diep OR 'tram flap'/exp OR tram OR 'transverse rectus abdominis musculocutaneous' OR 'rectus abdominis myocutaneous' OR siea OR 'superficial inferior epigastric artery' OR tug OR 'transverse upper gracilis' OR pap OR 'profunda artery perforator' OR gap OR 'gluteal artery perforator' OR 'latissimus dorsi flap'/exp OR 'latissimus dorsi' OR 'ld flap' OR implant OR 'tissue expander' OR autologous OR flap* OR reconstruction)
AND
('sensation'/exp OR 'sensory system'/exp OR 'sensory reinnervation'/exp OR neurotization OR 'nerve coaptation' OR 'nerve regeneration'/exp OR 'nerve graft'/exp OR 'nerve transfer' OR 'nerve repair' OR 'reinnervation'/exp OR 'sensory restoration' OR 'neural coaptation' OR 'neuroplasticity'/exp OR sensibility OR 'sensory recovery' OR 'nerve preservation' OR 'nerve conduit' OR 'nerve allograft' OR resensitization OR neurotis*:ti,ab OR neurotiz*:ti,ab OR neurorraph*:ti,ab OR reinnervat*:ti,ab OR sensation)

Web of Science:

TS=("Breast Neoplasms" OR "Mammaplasty" OR "Post-Mastectomy" OR Breast*)
AND
TS=("DIEP" OR "Deep Inferior Epigastric Perforator" OR "TRAM" OR "Transverse Rectus Abdominis Musculocutaneous" OR "Rectus Abdominis Myocutaneous" OR "SIEA" OR "Superficial Inferior Epigastric Artery" OR "TUG" OR "Transverse Upper Gracilis" OR "PAP" OR "Profunda Artery Perforator" OR "GAP Flap" OR "Gluteal Artery Perforator" OR "Latissimus Dorsi" OR "LD Flap" OR "Implant" OR "Breast Reconstruction" OR "Mastectomy" OR "Tissue Expander" OR "Autologous" OR "Flap" OR "Reconstruction")
AND
TS=("Sensation" OR "Sensory Reinnervation" OR "Neurotization" OR "Nerve Coaptation" OR "Nerve Regeneration" OR "Nerve Graft" OR "Nerve Transfer" OR "Nerve Repair" OR "Reinnervation" OR "Sensory Restoration" OR "Neural Coaptation" OR "Neuroplasticity" OR "Sensibility" OR "Sensory Recovery" OR "Nerve Preservation" OR "Nerve Conduit" OR "Nerve Allograft" OR "Resensitization" OR Neurotis* OR Neurotiz* OR Neurorraph* OR Reinnervat*)
