# Supplementary material for: Sensory Outcomes and Neurotization Techniques Following Mastectomies: A Comprehensive Systematic Review
Source: Cancers (Basel). 2026 Mar 24;18(7):1052. doi: 10.3390/cancers18071052 (PMC13072177; doi:10.3390/cancers18071052)
Supplement: Supplementary file 1 [file cancers-18-01052-s001.zip › Supplemental Table S1.pdf]

Supplemental Table S1: Risk-of-bias assessment of studies included in analysis

**Non-randomized (Newcastle-Ottowa Scale)***	**Selection (max 4)**	**Comparability (max 2)**	**Outcome (max 3)**	**Total quality score (max 9)**
Slezak et al. (1992)[8]	★★		★★	4
Yano et al. (1998)[39]	★★	★	★★	5
Blondeel et al. (1999)[33]	★★		★★	4
Blondeel et al. (1999)[42]	★★		★★	4
Isenberg et al. (2002)[26]	★★		★★	4
Yano et al. (2002)[41]	★★		★★★	5
Isenberg et al. (2004)[25]	★★		★★	4
Yap et al. (2005)[38]	★★	★	★★	5
Puonti et al. (2011)[36]	★★	★	★★	5
Mori et al. (2011)[35]	★★	★	★★	5
Spiegel et al. (2013)[40]	★★	★★	★★	6
Magarakis et al. (2013)[27]	★★	★	★★	5
Puonti et al. (2017)[53]	★★	★★	★★	6
Puonti et al. (2017)[54]	★★	★	★★	5
Cornelissen et al. (2018)[32]	★★	★★	★★	6
Beugels et al. (2019)[30]	★★	★★	★★	6
Peled et al. (2019)[59]	★		★★	3
Bijkerk et al. (2020)[31]	★★	★★	★★	6
Djohan et al. (2020)[58]	★★	★★	★★	6
Beugels et al. (2021)[28]	★★	★★	★★	6
Beugels et al. (2021)[34]	★★	★★	★★	6
Momeni et al. (2021)[44]	★★	★	★★	5
Tevlin et al. (2021)[45]	★★		★★★	5
Carrau et al. (2022)[46]	★★	★	★★	5
Huang et al. (2022)[49]	★★	★★	★★	6
Djohan et al. (2023)[43]	★★	★★	★★	6
Lu Wang et al. (2023)[51]	★★	★★	★★	6
Lu Wang et al. (2023)[52]	★★	★★	★★	6
Peled et al. (2023)[57]	★	★	★★	4
Zhang et al. (2024)[56]	★★	★	★★	5
Chang et al. (2024)[61]	★★	★	★★	5
Black et al. (2024)[48]	★★	★★	★★	6
Zhang et al. (2025)[24]	★★	★★	★★	6
Zhang et al. (2025)[50]	★★	★★	★★	6
Shyu et al. (2025)[60]	★★	★★	★★	6

**Randomized Trials (Cochrane Collaboration's tool)****	**Random seq. generation**	**Allocation concealment**	**Blinding of participants/personnel**	**Incomplete outcome data**	**Selective reporting**	**Other**
Temple et al. (2006)[37]	Low risk of bias	Low risk of bias	Low risk of bias	Low risk of bias	Low risk of bias	Low risk of bias
Temple et al. (2009)[55]	Low risk of bias	Low risk of bias	Low risk of bias	Low risk of bias	Low risk of bias	Low risk of bias
Bubberman et al. (2024)[29]	Low risk of bias	Low risk of bias	Low risk of bias	Unclear risk	Low risk of bias	Low risk of bias
Juan et al. (2024)[62]	Low risk of bias	Low risk of bias	Unclear risk	Low risk of bias	Low risk of bias	Low risk of bias

* Wells GA, Shea B, O'Connell D, et al. The Newcastle-Ottawa Scale (NOS) for assessing the quality of nonrandomised studies in meta-analyses. The Ottawa Hospital Research Institute. http://www.ohri.ca/programs/clinical_epidemiology/oxford.asp

** Higgins JPT, Altman DG, Gøtzsche PC, Jüni P, Moher D, Oxman AD, Savović J, Schulz KF, Weeks L, Sterne JA. The Cochrane Collaboration's tool for assessing risk of bias in randomized trials. *BMJ*, 2011; 343:d5928. doi: https://doi-org.proxy.uchicago.edu/10.1136/bmj.d5928
